# CesA6 and PGIP2 Endocytosis Involves Different Subpopulations of TGN-Related Endosomes

**DOI:** 10.3389/fpls.2020.00350

**Published:** 2020-03-27

**Authors:** Monica De Caroli, Elisa Manno, Carla Perrotta, Giulia De Lorenzo, Gian-Pietro Di Sansebastiano, Gabriella Piro

**Affiliations:** ^1^Department of Biological and Environmental Sciences and Technologies (DiSTeBA), University of Salento, Lecce, Italy; ^2^Dipartimento di Biologia e Biotecnologie “Charles Darwin”, Sapienza Università di Roma, Rome, Italy

**Keywords:** endocytosis, endosome, TGN, cellulose synthase, polygalacturonase inhibitor protein, traffic’s chemical inhibitors, SYP51

## Abstract

Endocytosis is an essential process for the internalization of plasma membrane proteins, lipids and extracellular molecules into the cells. The mechanisms underlying endocytosis in plant cells involve several endosomal organelles whose origins and specific role needs still to be clarified. In this study we compare the internalization events of a GFP-tagged polygalacturonase-inhibiting protein of *Phaseolus vulgaris* (PGIP2-GFP) to that of a GFP-tagged subunit of cellulose synthase complex of *Arabidopsis thaliana* (secGFP-CesA6). Through the use of endocytic traffic chemical inhibitors (tyrphostin A23, salicylic acid, wortmannin, concanamycin A, Sortin 2, Endosidin 5 and BFA) it was evidenced that the two protein fusions were endocytosed through distinct endosomes with different mechanisms. PGIP2-GFP endocytosis is specifically sensitive to tyrphostin A23, salicylic acid and Sortin 2; furthermore, SYP51, a tSNARE with interfering effect on late steps of vacuolar traffic, affects its arrival in the central vacuole. SecGFP-CesA6, specifically sensitive to Endosidin 5, likely reaches the plasma membrane passing through the *trans* Golgi network (TGN), since the BFA treatment leads to the formation of BFA bodies, compatible with the aggregation of TGNs. BFA treatments determine the accumulation and tethering of the intracellular compartments labeled by both proteins, but PGIP2-GFP aggregated compartments overlap with those labeled by the endocytic dye FM4-64 while secGFP-CesA6 fills different compartments. Furthermore, secGFP-CesA6 co-localization with RFP-NIP1.1, marker of the direct ER-to-Vacuole traffic, in small compartments separated from ER suggests that secGFP-CesA6 is sorted through TGNs in which the direct contribution from the ER plays an important role. All together the data indicate the existence of a heterogeneous population of Golgi-independent TGNs.

## Introduction

Endocytosis is a dynamic and complex cellular process required for the uptake of extracellular molecules, the regulation of plasma membrane protein and lipid turnover. After internalization, the endocytic vesicles are either fused with vacuoles or recycled back to the plasma membrane. In the last 30 years, endocytosis in plant cells has been demonstrated to be similar to the process in animal cells with pathways mediated by clathrin-dependent and clathrin-independent microdomain-associated endocytic mechanisms (Chen et al., 2011; Paez Valencia et al., 2016).

The clathrin-mediated endocytosis (CME) is the major route in plant cells and is evolutionary conserved, appearing similar to the animal model. The initiation of CME depends on three components: lipids, cargo and adaptor proteins and it is viewed as a highly coordinate step-wise process consisting of nucleation, cargo selection, vesicle coat assembly, scission and vesicle uncoating (McMahon and Boucrot, 2011).

A clathrin-independent pathway, defined as membrane microdomain-associated endocytosis, has been characterized in plants (Malinsky et al., 2013). Specific plasma membrane microdomains, enriched in sterols and sphingolipids, are involved in this internalization. The proteins flotillin and remorin, are involved and are considered the two marker proteins for these membrane microdomains (Wang et al., 2013; Fan et al., 2015). The *Arabidopsis* PIP2;1 (Li et al., 2011), AMT1;3 (Wang et al., 2013) and, in salt stress condition, RbohD (respiratory burst oxidase homolog D) (Hao et al., 2014) as well as the tomato sucrose transporter SISUT2 interacting proteins (Bitterlich et al., 2014) may be internalized into the cell via the membrane microdomain-associated endocytosis. It seems that microdomain-associated endocytosis occurs after protein clustering also in responses to extracellular stimuli (Katritch et al., 2013). Many plasma membrane proteins are present in monomeric form and are endocytosed via clathrin-mediated endocytosis but, in response to stress conditions, some of them form pre-dimers or dimers to subsequently assemble into clusters internalized via membrane microdomain-associated endocytic pathways. Probably, the cluster formation and clathrin-independent endocytosis provide an efficient mechanism to control the activity of membrane proteins in response to environmental changes (Fan et al., 2015).

A well accepted model indicates that the endocytic cargo internalized by clathrin-dependent or -independent endocytosis are always delivered to the *trans*-Golgi network (TGN), identified also as the early endosome (TGN/EE) (Dettmer et al., 2006; Lam et al., 2007; Viotti et al., 2010). Therefore, endocytosis and exocytosis intersect in TGN, though in different domains, as demonstrated with ECHIDNA and TRAPPII (Transport Protein Particle II) tethering complex *Arabidopsis* mutants. The proteins are in close proximity in the TGN in interphase cells but their localization diverges in dividing cells showing a dynamic localization in TGN subdomains related to specific and distinct functions (Gendre et al., 2011; Ravikumar et al., 2018). Such TGN subdomains may also differ for the contribution of different membrane traffic routes, in particular from Endoplasmic Reticulum (ER). For example, a direct traffic from ER to TGN has been suggested for the proteins AtRMR1 and AtRMR2 (Occhialini et al., 2016) and AtNIP1.1 (Barozzi et al., 2019).

The degradation pathway involves additional organelles, such as multivesicular bodies/late endosomes (MVBs/LE) and lytic vacuoles. Cargoes destined to degradation are trapped in the internal vesicle system of MVBs (Cui et al., 2018). The delivery of plasma membrane proteins/lipids to vacuoles requires previous monoubiquitination, which is the signal for the endosomal sorting complex required for transport (ESCRT) to the degradation pathway (Herberth et al., 2012). In plants, there are ESCRT-I, ESCRT-II and ESCRT-III involved in vacuolar degradation, and nine TOL (TOM1-LIKE) genes, which may be the functional equivalent of ESCRT-0 (Xie et al., 2019). ESCRT I and II recognize and concentrate ubiquitinated cargoes within EEs, preventing their recycling to the plasma membrane, ESCRT III and ESCRT-associated proteins play a role in EE membrane invagination, determining the inner morphology of MVBs (Raiborg and Stenmark, 2009). Therefore ESCRT-mediated sorting of cargo destined for degradation occurs in TGN/EE and it is hypothesized that MVBs, representing the late endosome (LE), originate from the maturation of specific TGN/EE domains. Several observations suggest that the trafficking from MVBs to vacuoles does not involve shuttle vesicles (Scheuring et al., 2011, 2012; Onelli and Moscatelli, 2013; Cui et al., 2016; Barozzi et al., 2019). Indeed, ultrastructural observations showed that MVBs directly fuse with vacuoles (Onelli and Moscatelli, 2013). The presence of an intermediate compartment, named late prevacuolar compartment (LPVC), was proposed to mature from MVBs and fuse to the vacuole (Gershlick et al., 2014); in fact, although the LPVC resembles MVBs morphologically, lipid and protein composition of the organelle-delimiting membrane seems to be modified during the maturation process.

When not directed to the degradation pathway, endocytosed proteins could be constitutively recycled to the PM; the TGN/EE takes part to this process which is crucial for the polar distribution of PM proteins. The ARF-GEF GNOM, that has recently shown to localize in Golgi apparatus (Naramoto et al., 2014), is involved in the constitutive recycling of auxin carrier PIN proteins (Luschnig and Vert, 2014) while small CesA compartments (SmaCCs) or microtubule-associated cellulose synthase compartments (MASCs) could be involved in the recycling of the PM cellulose synthase complex (Crowell et al., 2009; Gutierrez et al., 2009).

The contribution of the ER membrane described by these reports may be so relevant to drastically alter TGN subdomains identity. This line of evidence supports the idea that endocytic vesicles could be sorted through distinct TGN subdomains or through completely different TGN populations: the Golgi-associated TGN (Ga-TGN), cisternae attached to the *trans*-side of Golgi, and the Golgi-independent TGN (Gi-TGN), detached free TGN cisternae (Uemura et al., 2014; Rosquete et al., 2018).

In this work, we have studied, in a heterologous system, the internalization events of two proteins undergoing endocytosis: CesA6 of *Arabidopsis thaliana* and PGIP2 of *Phaseolus vulgaris*. *At*CesA6 is a subunit of the Cellulose Synthase Complex (CSC), constantly recycled from the PM to a dedicated internal compartment (Paredez et al., 2006; Crowell et al., 2009). PGIP2 is an apoplastic protein that inhibits the activity of microbial endopolygalacturonases (PGs). We have previously shown that PGIP2 of *P. vulgaris*, transiently expressed as fluorescent fusion protein in tobacco leaf protoplasts, after secretion in the regenerating cell wall, undergoes internalization in the absence of its natural fungal interactor (De Caroli et al., 2011a, b). These two proteins follow very diverse pathways and are then good candidates to be used as markers of separated TGN/EE subpopulations. We compared the sorting pathway of the tagged variants of CesA6 and PGIP2 applying several pharmacological treatments and the interfering effect of a SNARE (SYP51) that, all together, revealed clear and important differences at the level of TGN/EE between the two pathways.

## Materials and Methods

### Plasmid Construction

Oligonucleotides used for cloning can be found in Supplementary Table 1. To construct the plasmid for the expression of secGFP-CesA6, *Sal*I and *Pst*I restriction sites were introduced in CesA6 coding sequence (TAIR: At5g64740) using the primer CesA6for and CesA6rev (Supplementary Table 1). The *Sal*I/*Pst*I fragment was inserted into a GFP-containing vector secGFP-PGIP2 (De Caroli et al., 2011a) substituting the PGIP2 coding sequence. A 2-kb fragment of the Arabidopsis CesA6 promoter upstream of the initiation codon was amplified by PCR with pCesA6-*Xho*I and pCesA6-*Bam*HI primers (Supplementary Table 1) containing *Xho*I and *Bam*HI restriction sites. It was cloned into the pGY vector (De Caroli et al., 2015) as a *Xho*I/*Bam*HI fragment, after the removal of the 35S promoter. The genomic DNA used for cloning the native promoter of CesA6 was extracted from 3-week-old Arabidopsis plants as previously described (Iurlaro et al., 2016; Lionetti et al., 2017). The construct was confirmed by sequencing (Eurofins Genomics^1^). SecGFP-CesA6 was inserted as an *Eco*RI/*Sac*I fragment into the plant binary vector pBIN19 (Di Sansebastiano et al., 2004; De Caroli et al., 2011a) named secGFP-CesA6 to evidence the control of an endogenous promoter. It was used both for *Agrobacterium*-mediated transient and stable transformation of tobacco.

51F-PGIP2-RFP/GFP and 51T-PGIP2-RFP double constructs were obtained from vectors described by De Benedictis et al. (2013) substituting AleuGFP *Eco*RI/*Eco*RI expression cassette with the PGIP2-RFP cassette derived from the pGY vector (De Caroli et al., 2015). Both double constructs are under the control of CaMV 35S promoter.

### Agrobacterium-Mediated Transient Transformation of Tobacco Leaf Epidermal Cells

The PGIP2-GFP/RFP (De Caroli et al., 2015), secGFP-CesA6, ST52-mCherry (De Caroli et al., 2011a), 51F-PGIP2-RFP/GFP, 51T-PGIP2-RFP and RFP:NIP1.1 (Barozzi et al., 2019), hereon indicated as RFP-NIP1.1, constructs were introduced into *Agrobacterium tumefaciens* (Strain GV3110). Agroinfiltration was performed in wild type (wt) *Nicotiana tabacum* or secGFP-CesA6 stably transformed leaves as described in Paris et al. (2010). To evaluate the Sp2 effect on the secretion of the tagged proteins, *dexSp2-14* transgenic plants (here named Sp2 plants) were infiltrated after induction of Sp2 expression as previously described (Geelen et al., 2002).

### Stable Transformation of Tobacco Plants

To obtain stable transformed tobacco plants, sterile leaves of wt tobacco plants grown in solid MS medium (Sigma-Aldrich^2^; salt medium; 30% sucrose; 0.8% Bacto-agar) were used as partially described in previous reports (Di Sansebastiano et al., 2004). The bacterial suspension of *A. tumefaciens* (GV3110 strain), transformed with secGFP-CesA6 construct with endogenous promoter, grown over night, were pelleted at 4000 rpm for 10 min and re-suspended in 30 ml of liquid MS medium. Several pieces of leaves were cut from different wt plants and were immersed in the solution containing the MS medium with the Agrobacterium in order to allow the infection. The infection time of the Agrobacterium on the leaf was 10 min. Subsequently, the Agrobacterium in excess was removed, eliminating the liquid in which it was re-suspended. The residues of bacterial suspension were removed with sterile filter paper twice. The infected leaf pieces were plated on a solid MSS medium (Solid MS medium, 1 mg/L BAP, pH 5.8) and incubated for 24 h at 25°C in the dark. The plates containing the infected leaf pieces were exposed to light at 25°C for 4 days. The fragments of infected leaves were washed with a solution of liquid MS medium containing 500 mg/L of Cefotaxime (Sigma-Aldrich^2^), in order to completely remove the excess of Agrobacterium. The leaflets of the infected leaves, thus washed, were transferred to MSSCK plates, MSS containing 400 mg/L Cefotaxime and 100 mg/L Kanamycin (Sigma-Aldrich^2^). The leaflets were transferred weekly to new MSSCK plates. The first calluses appeared after 5 weeks of processing. After 2 weeks the appeared regenerating shoots were transferred on new plates containing solid MS medium and Kanamycin (100 mg/L). Five independently transformed plants were selected for their stronger fluorescent signal and reproduced to the third generation. Plantlets were analyzed at microscopically and showed the same fluorescent pattern. Transgenic lines from 1 to 5 were used for the experiments showing no differences.

### Drug Treatments

All the drug treatments were performed by inhibitor infiltration in transiently transformed leaf. The final concentration of the inhibitor used were: 350 μM for tyrphostin A23 (TyrA23; Sigma-Aldrich^2^), 100 μM for salycid acid (SA; Sigma-Aldrich^2^), 2 μM for concanamycin A (ConA; Sigma-Aldrich^2^), 3 μM for wortmannin (Wm; Sigma-Aldrich^2^), 20 μM for Sortin 2 (ChemBridge Corporation^3^), 50 μM for Endosidin 5 (ES5; ChemBridge Corporation^3^).

To test BFA (Sigma-Aldrich^2^) effect on endocytosis it was necessary to distinguish between newly synthesized and endocytosed proteins, so leaves transiently expressing PGIP2-GFP and stably expressing secGFP-CesA6 were infiltrated with cycloheximide (CHX; Sigma-Aldrich^2^) to the final concentration of 300 μM. The same procedure was applied to ConA treatment on secGFP-CesA6 accordingly to Dettmer et al. (2006). The leaf discs (1 cm diameter) taken from treated leaves were incubated by immersion in water containing 10 μM of FM4-64 dye (Invitrogen Molecular Probes^4^) before treatment with BFA. In the case of co-expression of secGFP-CesA6 with ST52-mCherry, CHX pre-treatment was not applied. Leaf discs were treated by immersion in solution with BFA at the final concentration of 100 μM and the induced fluorescent pattern was compared to the control conditions. Images were collected at the indicated times of incubation.

### Confocal Laser Scanning Microscopy

Transiently and stably transformed plants were examined as previously described (De Caroli et al., 2014) using a confocal laser scanning microscope LSM 710 Zeiss (ZEN Software, GmbH, Germany). GFP was detected within the short 505–530 nm wavelength range, assigning the green color, RFP within 560–615 nm assigning the red color. Excitation wavelengths of 488 and 543 nm were used. The laser power was set to a minimum and appropriate controls were made to ensure there was no bleed-through from one channel to the other. Images were processed using Adobe Photoshop 7.0 software (Mountain View, CA, United States).

### Data Analyses

The quantitative evaluation of drugs’ effect on the fluorescent pattern of secGFP-CesA6 and PGIP2-GFP in the epidermal cells of leaves was carried out by counting in different equivalent areas, the number of intracellular compartments classified in three different populations. The compartments with dimensions less than 0.83 μm, those with dimensions between 0.83 and 1.56 μm and those with dimensions greater than 1.56 μm were evaluated in confocal images of epidermal cells of control and drug-treated leaves. When a complete change of pattern was observed statistical analysis was not performed (PGIP2-GFP plus TyrA23, SA, Wm, Sortin 2).

Results are presented as the mean value ± standard deviation (SD) of *n* independent replicate experiments as reported in each Figures. One-way ANOVA was performed. T-student method was applied to establish significant difference between means (*p* < 0.005). All statistical comparisons were performed using SigmaStat version 11.0 software (Systat Software Inc., Chicago, IL, United States).

## Results

### PGIP2-GFP but Not SecGFP-CesA6 Internalization Occurs via Clathrin-Mediated Endocytosis

Previous studies using fluorescent tags showed that PGIP2 (PGIP2-GFP) and CesA6 (secGFP-CesA6) reach the plasma membrane through independent sorting pathways. Both of them can be then internalized and the involved mechanisms were here studied with a comparative approach.

The sorting of the two proteins confirmed what was previously described in other studies. After secretion in the cell wall (Supplementary Figure 1A), PGIP2-GFP was endocytosed (Supplementary Figure 1B) and targeted to the vacuole (Supplementary Figure 1C) because its natural extracellular ligand, the fungal endopolygalacturonase (PG) was missing (De Caroli et al., 2011a, b). SecGFP-CesA6 was cycled between plasma membrane and various subcellular compartments (Desprez et al., 2007; Gutierrez et al., 2009). In tobacco cells it showed a patchy distribution on the plasma membrane (Supplementary Figures 2A–C) and accumulation in punctate compartments, likely Golgi bodies and small intracellular complexes closely associated to microtubules known as MASCs (Microtubule-Associated cellulose Synthase Compartments) (Supplementary Figures 2D–F). These compartments were distinguishable from endocytic compartments labeled by FM4-64 (Supplementary Figures 2G–I). Persistence over time of this pattern is consistent with recycling from the plasma membrane through endocytosis (Supplementary Figures 2J–L).

**FIGURE 1:**
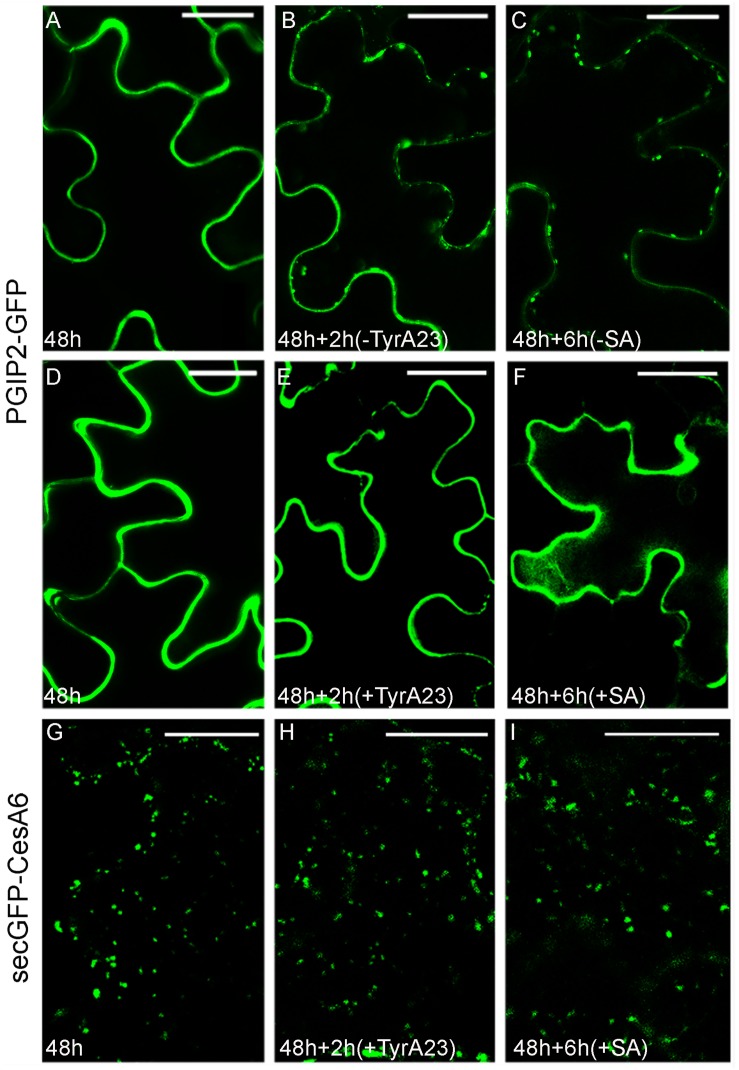
Tyrphostin A23 (TyrA23, 350 μM) and salicylic acid (SA, 100 μM) treatments on tobacco leaf epidermal cells transiently transformed with PGIP2-GFP **(A–F)** and secGFP-CesA6 **(G–I)**. **(A)** PGIP2-GFP fluorescent pattern at 48 h after transformation showed mainly CW and few endosomes (see also Supplementary Figure 1D); **(B)** after additional 2 h, the time corresponding to treatment without TyrA23, more endosomes can be observed; **(C)** after 6 h, the time corresponding to SA treatment, endosomes number increases; **(D)** PGIP2-GFP fluorescent pattern at 48 h after transformation; **(E)** after additional 2 h of TyrA23 treatment only CW appears fluorescent; **(F)** similarly, after 6 h of SA treatment, the fluorescence pattern does not change. **(G)** SecGFP-CesA6 fluorescent pattern at 48 h after transformation is characterized by punctate compartments; **(H)** after additional 2 h of TyrA23 treatment or **(I)** 6 h of SA treatment there are no evident changes in the fluorescent pattern of secGFP-CesA6. Statistic is provided in Supplementary Figure 3. Scale bars: 20 μm.

To test the existence of common steps of endocytosis for PGIP2-GFP and secGFP-CesA6, we used tyrphostin A23 (TyrA23), an inhibitor of the clathrin-mediated endocytosis (Ortiz-Zapater et al., 2006), previously shown to affect PGIP2-GFP internalization in tobacco protoplasts (De Caroli et al., 2011a). As expected, also in tobacco epidermal leaf cells PGIP2-GFP internalization from the cell wall to the endosomes (Figures 1A–C) and to the large central vacuole (Supplementary Figure 1C) was drastically inhibited (Figure 1E) by a 2 h treatment with 350 μM TyrA23 respect to the relative control (Figure 1B). It is known that this drug acts through the acidification of cytoplasm (Dejonghe et al., 2016) so we also tested salicylic acid (SA, 100 μM) that, should interfere with the clathrin-mediated endocytosis (Du et al., 2013) too. After 6 h of treatment with 100 μM SA, PGIP2-GFP fluorescence was totally retained into the cell wall (Figure 1F) respect to untreated epidermal cells (Figure 1C). When TyrA23 or SA were tested on secGFP-CesA6, the fluorescent pattern of the chimera was not altered: intracellular punctate compartments of different size were visible in all analyzed conditions (Figures 1G–I). Since secGFP-CesA6 is recycled in different organelles and the mis-sorting to an aberrant destination cannot be observed, the percentage of punctate compartments in the range of 0.83–1.56 μm was evaluated and found to be variable in a not statistically significant way (Supplementary Figure 3). Two different drugs considered as inhibitors of clathrin-mediated endocytosis were then able to affect PGIP2-GFP’s but not secGFP-CesA6’s sorting demonstrating differences in the early stages of internalization.

### PGIP2-GFP and SecGFP-CesA6 Endocytosis Diverge Also at the Level of Late Endosome

To further characterize PGIP2-GFP and secGFP-CesA6 endocytic pathway steps, we tested the effect of wortmannin (Wm) and concanamycin A (ConA). Wm is a phosphatidylphosphate-3-kinase inhibitor specifically affecting several traffic events; at low concentrations, it affects PVC/MVB/LE fusion to the vacuole (Foissner et al., 2016). ConA acts on V-ATPase activity in the TGN blocking both the trafficking to the plasma membrane and the endocytosis of FM4-64 from the TGN/EE to the vacuole (Dettmer et al., 2006).

**FIGURE 2:**
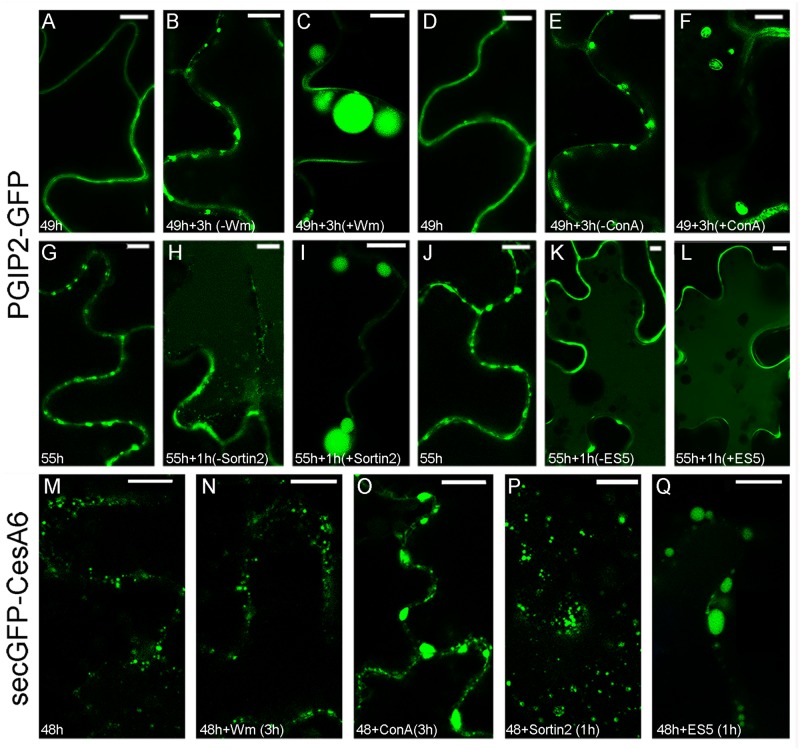
Effect of chemical treatments on tobacco epidermal leaf cells transiently transformed with PGIP2-GFP **(A–L)** and secGFP-CesA6 **(M–Q)**. **(A)** Fluorescent pattern of PGIP2-GFP 49 h after infiltration; **(B)** PGIP2-GFP after additional 3 h; **(C)** PGIP2-GFP after 3 h of Wm (3 μM) treatment, the pattern is characterized by enlarged endosomes similar to provacuoles; **(D)** fluorescent pattern of PGIP2-GFP 49 h after infiltration in independent experiment; **(E)** PGIP2-GFP after additional 3 h; **(F)** after 3 h of ConA (3 μM) treatment, the drug induces enlarged compartments in which multiple membranes can be observed, as expected in MVBs (statistic in Supplementary Figure 4); **(G)** PGIP2-GFP 55 h after infiltration; **(H)** after additional 1 h, central vacuole starts to be labeled by PGIP2-GFP fluorescence; **(I)** after 1 h of Sortin2 (20 μM) treatment, the PGIP2-GFP pattern is characterized by enlarged endosomes; **(J)** PGIP2-GFP 55 h after infiltration; **(K)** PGIP2-GFP after additional 1 h; **(L)** after 1 h ES5 (50 μM) treatment, PGIP2-GFP fluorescence pattern does not change; **(M)** fluorescent pattern of secGFP-CesA6 48 h after infiltration shows intracellular punctate compartments; **(N)** after additional 3 h in the presence of Wm (3 μM), no apparent effect on secGFP-CesA6 pattern is observed; **(O)** after 3 h of ConA (3 μM) treatment secGFP-CesA6 shows enlargement of the labeled compartments; **(P)** after 1 h of Sortin2 (20 μM) treatment, secGFP-CesA6 fluorescent pattern does not change; **(Q)** after 1 h of ES5 (50 μM) treatment, secGFP-CesA6 is visible in enlarged compartments. Statistics for secGFP-CesA6 treatments is provided in Supplementary Figure 5. Scale bars: 10 μm.

The Wm drug (3 μM for 3 h) significantly modified the endocytic pathway of PGIP2-GFP that, instead of evolving from a distribution in the cell wall and endosomes (Figures 2A,B) to the central vacuole (Supplementary Figure 1), was almost completely blocked in enlarged endosomal compartments of the size of small vacuoles (Figure 2C) with the simultaneous decrease of fluorescence in the central vacuoles (quantification not shown). The change of morphology was so extreme that statistical evaluation was not necessary. Also ConA treatment (2 μM for 3 h) affected PGIP2-GFP accumulation in the vacuole inducing the enlargement of intermediate compartments (Supplementary Figure 4) that also changed morphology showing a more evident organization in MVB (Figure 2F). An alteration of PGIP2-GFP distribution, more similar to the pattern induced by Wm, was caused by treatment with Sortin 2 (20 μM for 1 h) (Figure 2I), a chemical compound that interferes with ESCRT components (Zouhar et al., 2004). Again, the change of morphology was so extreme that statistical evaluation was not necessary. On the contrary, no effect was observed on PGIP2-GFP after treatment with Endosidin 5 (ES5; 50 μM for 1 h) (Figure 2E) that should block proteins recycling from plasma membrane (Li et al., 2012). The PGIP2-GFP sensitivity to Wm and Sortin 2 demonstrated its transition through PVC/MVB/LE.

The fluorescent pattern of secGFP-CesA6 (Figure 2M) suffered the effects of the drugs differently. It was not affected by the Wm treatment (Figure 2N), whereas it was altered after ConA treatment, with the significant enlargement (Supplementary Figure 5) of compartments labeled by secGFP-CesA6 (Figure 2O). Moreover, it was not affected by Sortin 2 (Figure 2P) and significantly affected (Supplementary Figure 5) by ES5 (Figure 2Q), which induced the formation of the typical Endosidin bodies (Drakakaki et al., 2011), enlarged compartments indicative of a block in the recycling of the protein. ConA was the only chemical inhibitor to modify the fluorescence pattern of both fluorescent proteins.

### PGIP2-GFP and SecGFP-CesA6 Are Differently Influenced by SYP51

**FIGURE 3:**
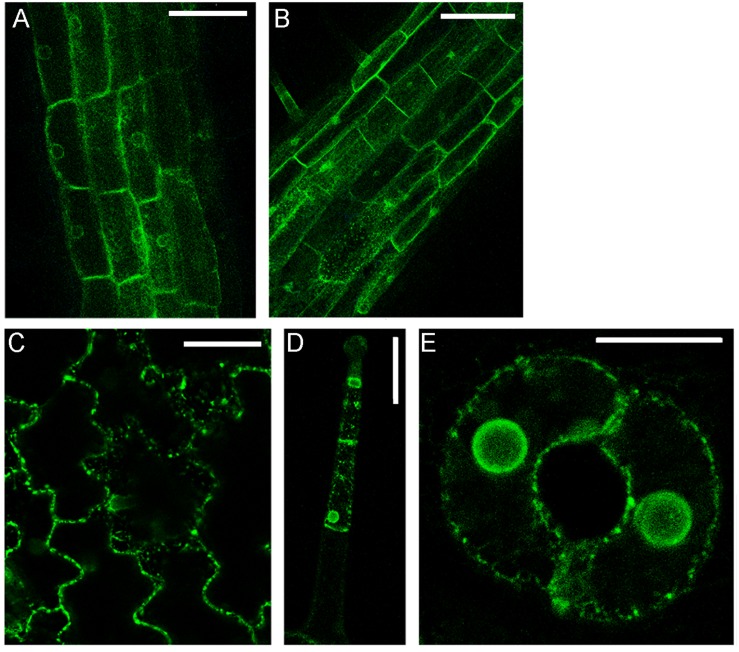
Details of confocal images of tissues from tobacco seedlings (line 1) stably expressing secGFP-CesA6: **(A)** hypocotyl epidermal cells; **(B)** root rizodermal cells; **(C)** leaf epidermal cells; **(D)** trichome cells and **(E)** guard cells of stomata. Scale bars: 100 μm **(A,B)**; 50 μm **(C,D)**; 20 μm **(E)**.

**FIGURE 4:**
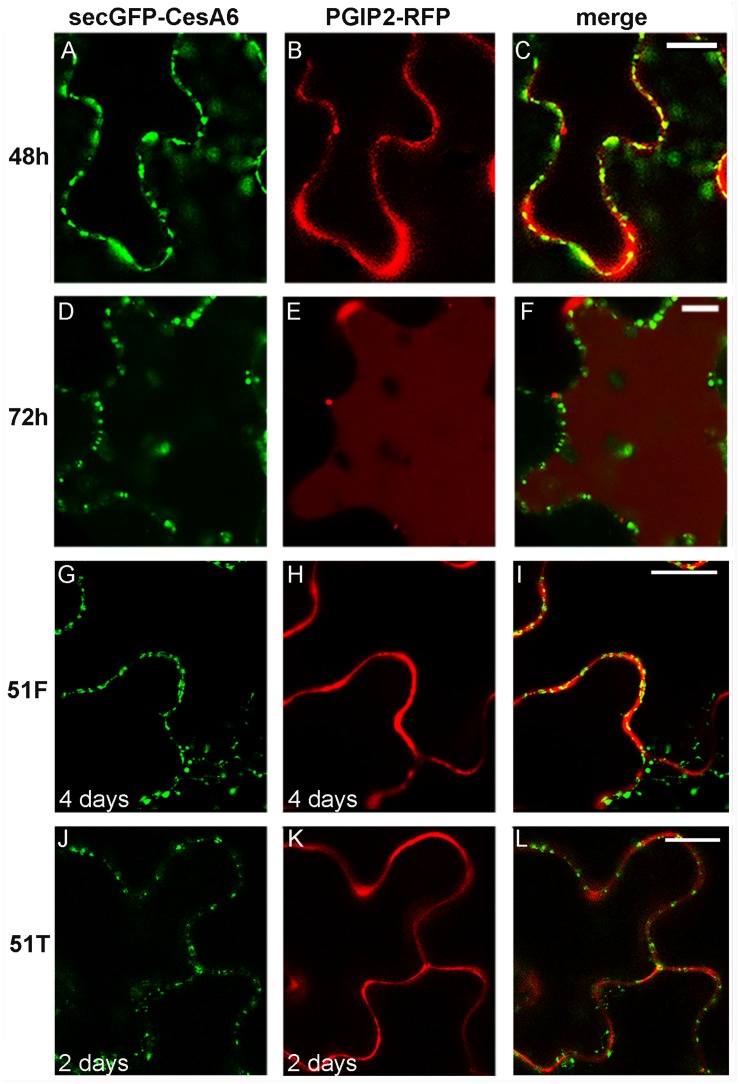
Confocal images of tobacco epidermal cells stably expressing secGFP-CesA6, line 1 **(A–F)** and line 2 **(G–L)**, transiently co-transformed with PGIP2-RFP to visualize canonical protein sorting progression over time **(A–F)** and transiently co-transformed with 51F-PGIP2-RFP **(G–I)** or 51T-PGIP2-RFP **(J–L)** to visualize SYP51 variants effect on sorting. After 48 h of expression, **(A)** secGFP-CesA6 green fluorescence; **(B)** PGIP2-RFP red fluorescence; **(C)** merge of fluorescent emissions. After 72 h **(D)** secGFP-CesA6 fluorescence; **(E)** PGIP2-RFP fluorescence; **(F)** merge of fluorescent emissions. Four days after transformation, **(G)** secGFP-CesA6 fluorescence; **(H)** PGIP2-RFP fluorescence during 51F co-expression; **(I)** merge of fluorescent emissions during 51F co-expression. Two days after transformation, **(J)** secGFP-CesA6 fluorescence; **(K)** PGIP2-RFP fluorescence during 51T co-expression; **(L)** merge of fluorescent emissions during 51T co-expression. Scale bars: 20 μm.

The simultaneous transient expression of secGFP-CesA6 and PGIP2-RFP altered their normal pattern of distribution in tobacco leaf epidermal cells, probably saturating ER and delaying endocytosis in general (not shown). In order to overcome this problem, we obtained stably transformed plants expressing secGFP-CesA6. The fluorescence pattern of secGFP-CesA6 was observed in different organs of transgenic T2 seedlings (leaf, hypocotyl and root). Hypocotyl, root and the aerial tissue’s cells showed a fluorescent distribution pattern identical to that observed in transiently transformed epidermal cells with a more evident labeling of the ER (Figures 3A–E). Transient transformation of these transgenic leaves with PGIP2-RFP allowed to observe that sorting of both proteins occurred with the same timing observed when the two constructs were expressed separately (Figures 4A–F). We then used this system to further investigate the internalization of the two co-expressed proteins, and in particular the involvement of specific SNAREs in the sorting pathways. With this aim, we analyzed the effect of the vacuole-related SNARE SYP51 and of the exocytosis-related SNARE SYP121 on PGIP2-GFP and secGFP-CesA6 trafficking.

**FIGURE 5:**
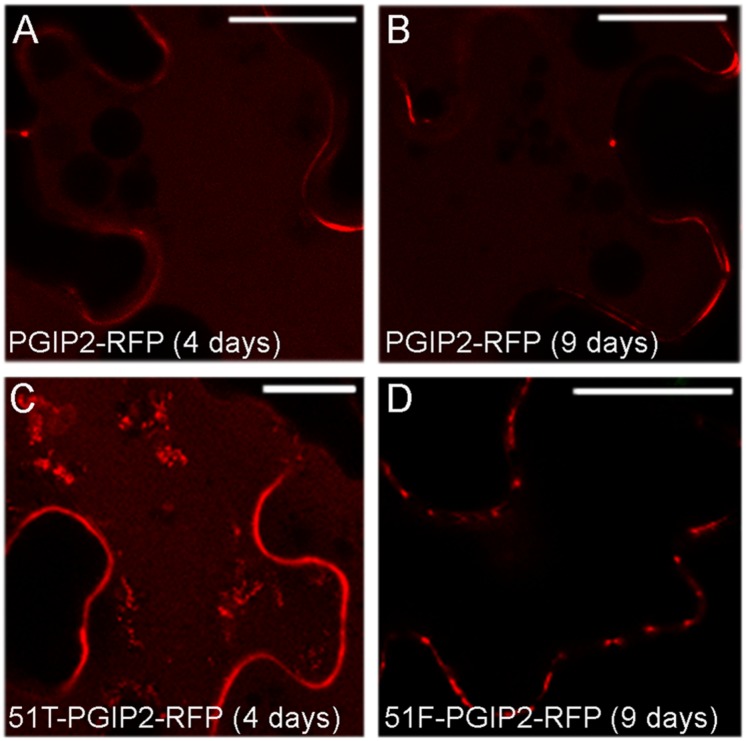
Confocal images of tobacco epidermal cells transiently expressing **(A)** PGIP2-RFP for 4 days; **(B)** PGIP2-RFP for 9 days; **(C)** 51T-PGIP2-RFP for 4 days; **(D)** 51F-PGIP2-RFP for 9 days. The co-expression of SYP51 variants (51F and 51T) affects PGIP2-RFP sorting. Scale bars: 20 μm.

SYP51 has a relevant role in both secretion and endosomes organization as shown by using both the entire native form (51F) and a dominant negative truncated protein form (51T), lacking the C-terminal *trans*-membrane domain (De Benedictis et al., 2013). The involvement of this SNARE in the sorting of PGIP2 and CesA6 was investigated using the consolidated approach based on the effect of 51F and 51T overexpression (Di Sansebastiano and Barozzi, 2017). Two constructs were produced to drive simultaneous expression of PGIP2-RFP and 51T or, alternatively, 51F, under the CaMV 35S promoter. The obtained constructs were, respectively, named 51T-PGIP2-RFP and 51F-PGIP2-RFP. These double constructs were used to agroinfiltrate leaves of the transgenic tobacco leaves stably expressing secGFP-CesA6, generating a triple transgene expression and a double fluorescent labeling. It was observed that the typical fluorescent pattern of secGFP-CesA6, consisting of a patchy labeled plasma membrane and intracellular compartments, was not altered by co-expression of either 51F or 51T (Figures 4G,I,J,L, respectively). Also PGIP2-RFP reached the cell wall with no alteration (Figures 4H,I,K,L); however, its internalization to the central vacuole was affected by the 51F co-expression (Figure 4). We verified the effect of 51T and 51F on the PGIP2 sorting to the vacuole also by transient expression in wild-type tobacco epidermal cells of 51T-PGIP2-RFP and 51F-PGIP2-RFP. The experiment confirmed that the protein internalization, that sorted fluorescence in the central vacuole after few days (Figures 5A,B), was weakly affected by 51T variant co-expression, but more clearly inhibited by 51F after long expression time (Figures 5C,D). In our experimental system we observed that 4 days after transformation the co-expression of 51T induced the persistence of labeling in intermediate compartments but the fluorescence arrived anyhow in the central vacuole (Figure 5C), on the contrary the co-expression of 51F completely blocked the fluorescent marker arrival in the central vacuole (Figure 5D).

We also tested the effect of a SNARE important for the exocytic post-Golgi pathway, using the dominant negative (DN) mutant of SYP121 (here named Sp2). It was previously shown that Sp2 did not affect PGIP2 secretion to the cell wall in tobacco protoplasts (De Caroli et al., 2011a). Transient expression of the two fluorescent proteins in epidermal cells of transgenic plants expressing Sp2 driven by a DEX inducible promoter (Geelen et al., 2002) showed that both of them normally reached the cell wall and the plasma membrane without suffering any inhibitory effect (Supplementary Figure 6).

### SecGFP-CesA6 Endocytosis and Sorting May Be Partially Golgi-Independent

Endocytosis of either CesA6 or PGIP2 tagged with GFP and the endocytic dye marker FM4-64 was compared by co-localizing fluorescence distribution in control conditions and during BFA treatment. It was previously shown that PGIP2-GFP was internalized in the same compartments labeled by FM4-64 (De Caroli et al., 2011a), on the contrary it was here observed that secGFP-CesA6 did not label these compartments (Supplementary Figures 2G–I).

**FIGURE 6:**
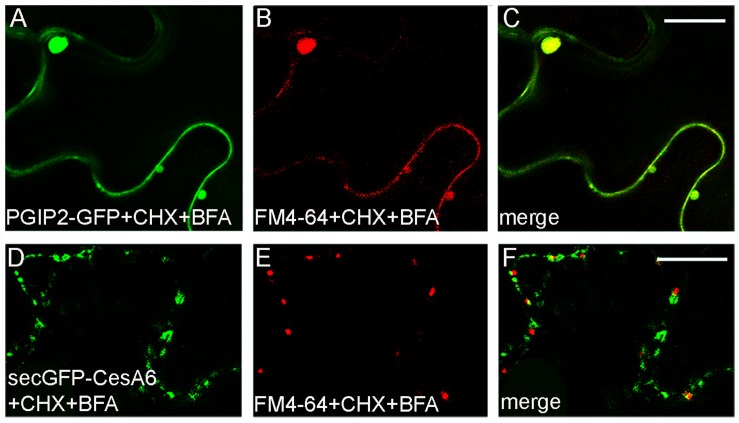
Tobacco epidermal leaf cells transiently expressing PGIP2-GFP **(A–C)** or secGFP-CesA6 **(D–F)** 2 h after infiltration with CHX (300 μM) followed by FM4-64 plus BFA (100 μM) treatments (30 min of leaf disc immersion). PGIP2-GFP and FM4-64 completely co-localized in BFA bodies **(A–C)** while the co-localization of secGFP-CesA6 BFA-induced compartments with the red dye is almost absent **(D–F)**. Scale bars: 20 μm.

In order to inhibit new proteins synthesis and favor the visualization of endocytic compartments with respect to compartments carrying newly synthesized marker for secretion, leaves transiently expressing PGIP2-GFP or secGFP-CesA6 were infiltrated with cycloheximide (CHX, 300 μM). The leaf discs taken from treated leaf were incubated by immersion with BFA (100 μM) in the presence of FM4-64. Both PGIP2-GFP and secGFP-CesA6 fluorescent pattern was altered by the appearance of larger compartments but we observed a complete co-localization with FM4-64 for PGIP2-GFP (Figures 6A–C); on the contrary the BFA-sensitive intracellular compartments labeled by secGFP-CesA6 did not co-localize with those labeled by the endocytic dye marker (Figures 6D–F).

**FIGURE 7:**
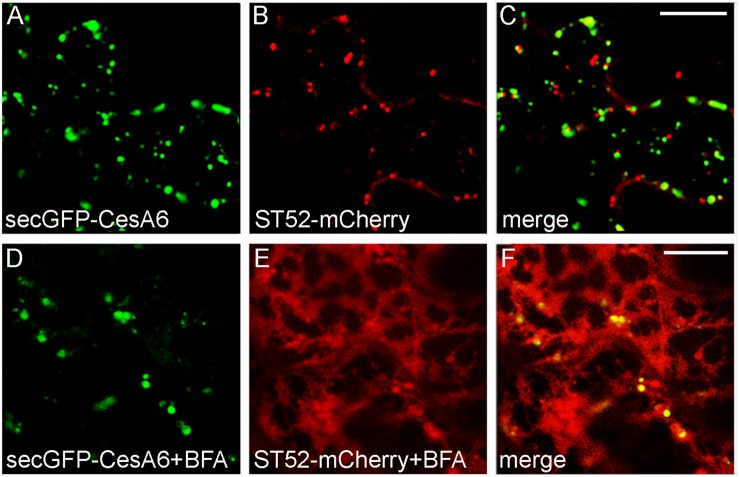
Epidermal leaf cells of tobacco plants (line 2) stably expressing secGFP-CesA6 **(A,C,D,F)** and transformed with ST52-mCherry **(B,C,E,F)** in control conditions **(A–C)** or treated with BFA (100 μM) **(D–F)**. SecGFP-CesA6 and the Golgi marker ST52-mCherry patterns show a very low overlap **(A–C)** also after BFA treatment (30 min of leaf disc immersion) **(D–F)**. Scale bars: 20 μm.

We also analyzed the BFA effect during co-expression of secGFP-CesA6 with the Golgi marker ST52-mCherry. The CSCs are thought to be assembled in the Golgi apparatus (Lampugnani et al., 2019) and we expected an extensive co-localization of secGFP-CesA6 with the Golgi marker; instead, co-localization with ST52-mCherry was surprisingly limited (Figures 7A–C). BFA treatment (100 μM) caused very distinctive effect on the two fluorescent proteins within the first 30 min of treatment. SecGFP-CesA6 was redistributed in aggregates and dotted structures but not in the ER (Figures 7D,F). ST52-mCherry suffered a completely different effect, being redistributed entirely in the ER (Figures 7E,F). The GFP labeling of typical BFA bodies was compatible with the aggregation of TGNs (Ritzenthaler et al., 2002) and tethering of MASCs (Gutierrez et al., 2009), while the ST52-mCherry labeling of ER was compatible with the re-distribution of Golgi-passing proteins due to COPII traffic inhibition (daSilva et al., 2004; Marti et al., 2010).

**FIGURE 8:**
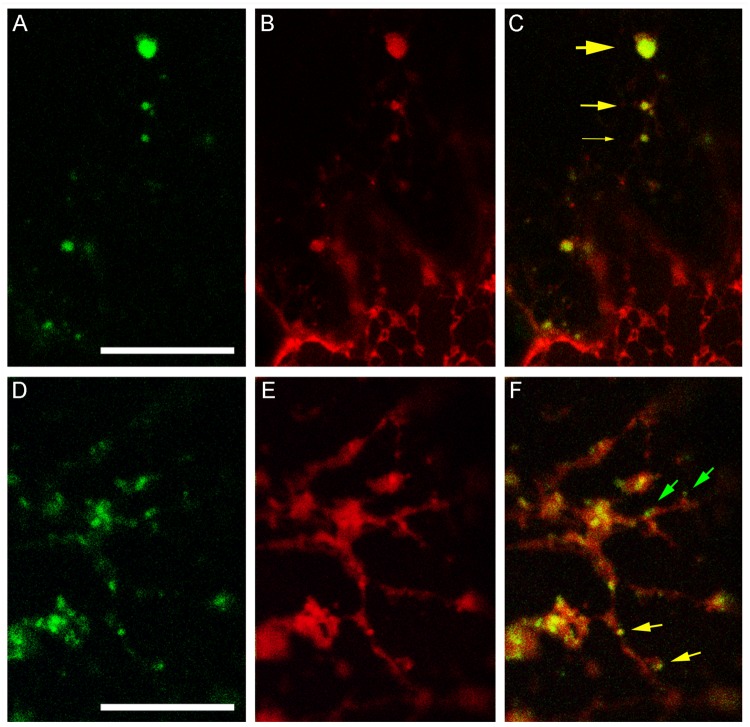
Confocal images of tobacco epidermal cells transiently co-expressing secGFP-CesA6 and RFP-NIP1.1. **(A–C)** secGFP-CesA6 (green fluorescence shown in panel **(A)** co-localizes with RFP-NIP1.1 (red fluorescence shown in panel **(B)** in compartments separated from the ER and variable in size evidenced by yellow arrows of different sizes **(C)**; **(D–F)** smaller fluorescent compartments can be distinguished in two categories if secGFP-CesA6 fluorescence **(D)** co-localize with RFP-NIP1.1 fluorescence **(E)**; green arrows indicate small compartments only labeled by secGFP-CesA6, yellow arrows evidence co-labeling by RFP-NIP1.1 **(F)**. Scale bars: 20 μm.

The different BFA-induced patterns of secGFP-CesA6 and ST52-mCherry prompted us to verify the co-localization of secGFP-CesA6 with RFP-NIP1.1, a marker of the direct ER-to-vacuole traffic that labels ER membranes and occasionally the tonoplast (Barozzi et al., 2019). We observed that the distribution of secGFP-CesA6 (Figures 8A,D) and RFP-NIP1.1 (Figures 8B,E) was different, since secGF-CesA6 was absent from ER were most of RFP-NIP1.1 was localized. However, the two fluorescent proteins showed a nearly complete co-localization in compartments separated from ER (Figure 8A) that represent a minor but a characteristic part of the fluorescence pattern of RFP-NIP1.1 (Figure 8B). The ER-independent compartments had different size (Figure 8C, yellow arrows of different size) but all showed co-labeling with the same proportion of the two markers. In addition to co-labeled compartments (Figure 8F, yellow arrows) it was also possible to observe, occasionally, very small compartments exclusively labeled by secGFP-CesA6 (Figure 8F, green arrows), possibly corresponding to Golgi dictiosomes.

## Discussion

In this study, we highlighted the diversity of endosomes involved in the internalization mechanisms of PGIP2 and CesA6. PGIP2 is a soluble cell wall protein of *Phaseolus vulgaris* that, transiently expressed and secreted in the apoplast, in absence of its natural ligand PG (endopolygalacturonase II of *Aspergillus niger*), can be endocytosed and sorted into the vacuole (De Caroli et al., 2011a, b, 2015). CesA6 is an *Arabidopsis thaliana* CSC subunit (Desprez et al., 2007; Persson et al., 2007; Bashline et al., 2014) sorted through several compartments: ER, Golgi, TGN, intracellular heterogeneous vesicles and the plasma membrane (Dettmer et al., 2006; Crowell et al., 2009, 2010; Zhang et al., 2016; Polko and Kieber, 2019). The sorting of all CesAs through the Golgi follows that of their regulatory proteins such as STELLO (Zhang et al., 2016). In particular, CSCs transit through peculiar microtubule-associated cellulose synthase compartments (MASCs also called SmaCCs) that seem to be involved, rather than in their secretion, in the internalization of CSCs (Crowell et al., 2009; Lampugnani et al., 2019) for their continuous recycling between the plasma membrane and TGNs. Because of their diversity, these proteins were selected as markers useful to evidence the intermediate compartments belonging to their different pathways.

We provide here the evidence that PGIP2 and CesA6 follow specific and alternative traffic routes during endocytosis (see Figure 1). The first distinction is based on susceptibility to TyrA23 and SA. TyrA23 was used expecting the inhibition of the CME (Ortiz-Zapater et al., 2006) but since it was recently shown that this drug may have secondary effects acidifying the cytoplasm (Dejonghe et al., 2016), we used a second drug supposed to act differently. Therefore, we tested salicylic acid (SA) that was shown to interfere with the CME (Du et al., 2013) too. As previously reported in tobacco leaf protoplasts (De Caroli et al., 2011a), we confirmed that PGIP2-GFP internalization was affected by these drugs, indicating that its sorting occurred via a CME. On the contrary, these molecules did not affect the internalization of secGFP-CesA6, pointing to endocytic events distinct from that of PGIP2 and likely clathrin-independent.

The mechanism of CSC internalization still remains obscure. It has been reported that, in response to environmental stresses, CSCs internalization occurs rapidly, shutting down cellulose synthesis. The dimension of CSCs is expected to interfere with the formation of the clathrin coat formation (Gutierrez et al., 2009; Crowell et al., 2010). However, the involvement of μ2, a subunit of the heterotetrameric CME adaptor protein 2 (AP2) complex, in CSC endocytosis has been reported using Arabidopsis mutant plant lines that show several general defects in the endocytic processes (Bashline et al., 2013, 2015). Since the endocytosis frequency of CesAs was very low in the mutants, Bashline and co-workers suggested that some, but not all, CesAs are endocytosed through a CME mechanism. In addition, it was hypothesized that the CSCs might dissociate into the weak fluorescence intensity CesA subunits, before being endocytosed. Although CME could not be evidenced, observing the CesA6/microtubule association (Supplementary Figure 1), we speculate that the MASC-depending recycling occurs at much higher frequency than the CME leading to CSC degradation in the vacuole. These distinctive dynamics in the subcellular trafficking that underpins the remodeling of the cellulose biosynthetic machinery has been well documented during the transition from the primary cell wall (PCW) to the secondary cell wall (SCW), associated to xylem vessel development (Watanabe et al., 2018). The modification of the CSC vesicle traffic modulates the removal of PCW CesAs for degradation in the lytic vacuole, while SCW CesAs are actively delivered (and recycled) at the cell plasma membrane (Watanabe et al., 2018).

Comparing the PGIP2 and CesA6 endocytic patterns, the difference between an internalization pattern targeted to the vacuole for degradation and a continuous recycling appeared quite evident. The PGIP2-GFP fluorescence, in absence of the protein ligand PG, completely disappeared from the wall filling progressively TGN/EE, LE, MVB, prevacuolar compartment (PVC) and finally the vacuole. On the other hand, in the same period of time secGFP-CesA6 fluorescence marked the plasma membrane, TGN/EE and MASCs.

The different response of the two protein markers to drugs active on specific post-Golgi traffic events has allowed us to evidence the different sorting steps. Wm and Sortin 2, both affecting TGN/EE-LE-vacuole trafficking (Zouhar et al., 2004; Foissner et al., 2016), significantly modify the endocytic pathway of PGIP2-GFP, which was almost completely blocked in enlarged endosomal compartments, likely related to MVB and PVC. The effect of Wm was evident and specific on transport of PGIP2 to the vacuole also at a concentration (3 μM) lower than that which affects other degrading traffic events (Di Sansebastiano et al., 2007; Ariani et al., 2018). On the contrary ES5, which specifically affects the PM recycling in plant and animal cells (Drakakaki et al., 2011; Li et al., 2012), did not influence PGIP2 vacuolar sorting. A completely different response was obtained with the same chemicals on secGFP-CesA6, which was insensitive to Wm and Sortin 2 but was clearly affected by ES5, with the formation of the typical Endosidin bodies (Drakakaki et al., 2011), thus blocking the recycling of the chimera in endosomes different from those labeled by PGIP2-GFP, and likely represented by MASCs (see Figure 2).

We also explored the involvement of SNAREs in the marker endocytosis. SYP51 is a Qc-SNARE with a targeting role (tSNARE) at the TGN and an interfering role (iSNARE) on tonoplast (De Benedictis et al., 2013). The tSNARE effect was proven through the overexpression of a truncated soluble protein variant, 51T, which acts as a dominant negative mutant, while the iSNARE effect on the last step of vacuolar sorting was proven through the overexpression of the SYP51 native form, 51F (De Benedictis et al., 2013; Di Sansebastiano and Barozzi, 2017). PGIP2-GFP and secGFP-CesA6 were regularly targeted, respectively, to the apoplast and the plasma membrane in the presence of both SYP51 variants, which, instead, differentially affected internalization of the two proteins. PGIP2 endocytosis was altered by co-expression with 51F, which blocked the protein in the apoplast for a longer time, while none of the steps involved in CesAs recycling was altered by SYP51 variants (see Figures 4, 5). These results indicated that the sorting of both protein markers to the cell wall and plasma membrane was clearly independent from SYP51 but vacuole sorting of PGIP2 was more affected than plasma membrane-TGN recycling for CesA6. This is not surprising since the sorting of CesA6 and the other CesA subunits to the vacuole, for the physiological protein turnover, is much less relevant than the recycling of CSC. Furthermore, the recycling seems to involve the entire CSC and needs specific compartments like MASCs, while the degradative endocytosis likely occurs for the single subunit, and is therefore much more difficult to highlight. We also tested the Qa-SNARE SYP121 involvement in the secretion of the markers and found that overexpression of the DN mutant (Sp2) did not affect either secGFP-CesA6 or PGIP2 (De Caroli et al., 2011a) sorting to the apoplast. In a previous study it was shown that also matrix polysaccharides sorting was not affected by SYP121 DN mutant (Leucci et al., 2007), our results then confirm that the regulation of cell wall deposition is controlled by different specific SNAREs.

The possibility that TGN is the predominant compartment shared by PGIP2 and CesA6 during their endocytic route was analyzed by using ConA (Figure 2) and BFA (Figures 6, 7). The response to ConA, known to block both the trafficking from TGN to the plasma membrane and the sorting of the endocytic marker FM4-64 from the TGN/EE to the vacuole (Dettmer et al., 2006; Li et al., 2012), was not determinant, since both PGIP2-GFP and secGFP-CesA6 were retained in endosomal enlarged compartments. The effect of ConA may occur on a regulatory target common to both markers’ sorting or, as suggested by the induction of a similar fluorescent patter (Figures 2C,H), on early step of sorting when the two markers share the same compartment. More specific indications were obtained with BFA. The drug shows dual intracellular effects: one at the TGN, leading to the formation of the BFA aggregates with the endosomal membranes, the other, at early Golgi level, consisting in the redistribution of Golgi membranes into the ER (Langhans et al., 2011). To highlight only the endocytosis of the two fusion proteins, focusing on TGNs, we reduced the interference due to the secretion events by blocking the synthesis of new proteins with CHX and then we compared the endocytosis of the two protein markers was compared to that of FM4-64. In our experimental condition, BFA determined the alteration of the intracellular compartments labeled by both proteins. However, PGIP2-GFP and FM4-64 fluorescence clearly overlapped in the same aggregated compartments while secGFP-CesA6 filled different compartments. As reported in Arabidopsis (Gutierrez et al., 2009), BFA, besides determining the formation of BFA bodies, also causes cortical accumulation and tethering of MASCs. The CesA6 labeled compartments, not co-localizing with FM4-64, could therefore be MASCs aggregated to TGNs and different from those labeled by PGIP2 and FM4-64. Thus, PGIP2 vacuole sorting and CesA recycling routes appeared not to share any post-Golgi intermediate compartment including TGNs/EE if this compartment has to be intended homogenous in its composition.

The existence of multiple and functionally diversified early/recycling endosomes (EE/REs), distinct from TGN/EE, is widely contemplated in plant cells. For example, the GNOM factor, which is BFA sensitive, regulates the recycling of PIN1 and PIN3 (Geldner et al., 2003; Ding et al., 2011) but is not involved in the recycling of AUX1, an auxin transporter, and PIN2 (Geldner et al., 2003; Kleine-Vehn et al., 2006).

The secretion of PGIP2-GFP in the apoplast has been previously well dissected showing a conventional secretion of the protein which unequivocally passes through the Golgi stacks (De Caroli et al., 2011a, 2015). The Golgi stacks have also been reported to be part of the CesA6 sorting (Desprez et al., 2007; Atanassov et al., 2009; Zhang et al., 2016; Lampugnani et al., 2019) but, when we used BFA in CesA6 tobacco transgenic plants and co-transformed with the Golgi marker ST52-mCherry, we observed an alteration effect since slightly decrease of the number of bright punctate labeled by secGFP-CesA6, but no redistribution in the ER and *cis*-Golgi was observed (Figures 7D–F). On the contrary, it was well visualized with the ST52-mCherry. The BFA induced patterns of two markers did not co-localized completely in merge image (Figures 7D,E), as they did not co-localized in absence of the fungal toxin (Figures 7A–C). The BFA effect on secGFP-CesA6 was not homogeneous in all cells and, with time (after more than 1 h of BFA treatment) the marker was partially redistributed in the ER membrane co-localizing with ST52-mCherry. Anyhow, in a vast majority of cases, the compartments labeled by the two markers do not overlap, indicating that the transition of CesA6 through the Golgi stacks may not be the only sorting pathway followed by the protein. The possibility to reach the TGN through an additional Golgi-independent pathway has been shown for RMR proteins, which homodimers and heterodimers can reach the TGN by-passing the Golgi (Occhialini et al., 2016, 2018).

To investigate the possibility of a direct traffic from the ER to the TGN, contributing to the characterization of heterogeneous populations of post-Golgi compartments we used RFP-NIP1.1, a protein marker of the unconventional vacuolar traffic that by-passes the Golgi. RFP-NIP1.1 labels ER membranes and intermediate compartments that are still poorly characterized (Di Sansebastiano et al., 2017; Goring and Di Sansebastiano, 2017; Barozzi et al., 2019). SecGFP-CesA6 and RFP-NIP1.1 showed a nearly complete co-localization in compartments separated from ER (Figure 8A), indicating an evident contribution of ER membrane in the diversification of compartments labeled by CesA6, downstream of the Golgi.

**FIGURE 9:**
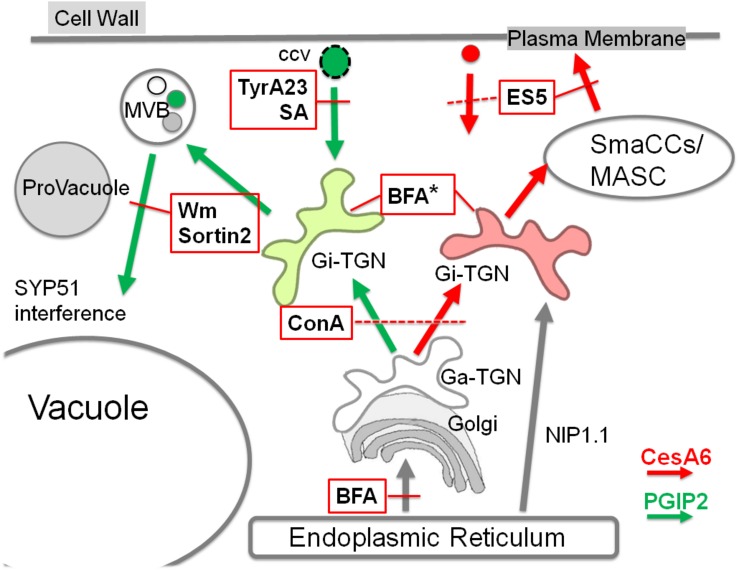
Schematization of endocytic traffic of PGIP2 (green arrows) and CesA6 (red arrows) affected by several small chemical molecules (abbreviations in the text). In the scheme only the inhibitory effects are reported, indicated with the intersection with red lines. Discontinuous red lines are used when the effect target is not well defined. In the case of BFA, in addition to the effect on ER export, an effect on TGNs morphology is indicated (*). We suggest the presence of different Golgi-independent TGNs (Gi-TGN), here evidenced by two different colors. They may differentiate during the maturation from the Golgi-associated form (Ga-TGN) because of a different contribution from endocytosis and from the ER membranes, characterized by the presence of NIP1.1. In addition to drug effects, also the interfering effect of the iSNARE SYP51 on endosome fusion to the vacuole is indicated.

In plant cells, the TGN is largely heterogeneous and comprises subpopulations that can be associated with the Golgi (Ga-TGNs/early TGNs) or independent from it (Gi-TGNs/late TGNs). Various TGN-associated proteins have been localized on overlapping, partially overlapping or separate TGNs, indicating the existence of multiple TGN subsets with distinct functions (Uemura et al., 2014). The association of ER membranes and endosomes, including TGN/EEs, has been already described and appears to be conserved in plant and mammalian cells (Friedman et al., 2013; Stefano et al., 2015). The ER network structure and movement are essential for the positioning and dynamics of endosomes and for endocytosis (Stefano et al., 2015, 2018; Renna et al., 2018); moreover, a heterogeneous population of ER-plasma membrane contact sites have been described (Pérez-Sancho et al., 2015; Siao et al., 2016; Stefano et al., 2018). We provide here further indications that the ER membrane contributes to shape separated membranous compartments related to TGNs with the potentiality to mature in different and functionally distinct compartments. This allows to explain the formation of CesA specific recycling endosomes (MASCs) separated from degrading endosomes (PVC, MVB) labeled by PGIP2. The model reported in Figure 9 suggests the presence of Gi-TGNs which assume their specific roles in degradative or recycling routes through the direct contribution of ER membranes, crucial for the differentiation of all endocytic compartments including diversified TGN/EE sub-populations.

## Data Availability Statement

All relevant data generated for this study are included in the article/Supplementary Material.

## Author Contributions

MD, GP, and G-PD designed the research. MD and EM performed the experiments. MD, EM, GP, CP, and G-PD analyzed data. MD and G-PD wrote the manuscript. GP, CP, and GD collaborated to writing-reviewing and editing of the manuscript.

## Conflict of Interest

The authors declare that the research was conducted in the absence of any commercial or financial relationships that could be construed as a potential conflict of interest.

## Footnotes

1 https://www.eurofinsgenomics.eu/

2 http://www.sigmaaldrich.com/

3 http://www.chembridge.com/

4 http://www.thermofisher.com
