# Supplementary material for: CesA6 and PGIP2 Endocytosis Involves Different Subpopulations of TGN-Related Endosomes
Source: Front Plant Sci. 2020 Mar 27;11:350. doi: 10.3389/fpls.2020.00350 (PMC7118220; doi:10.3389/fpls.2020.00350)
Supplement: Supplementary file 1 [file Data_Sheet_1.pdf]

Supplementary Table 1. Primers used for secGFP-CesA6 construction

secGFP-CesA6 constuction	
Name	Sequences (5'-3')
CesA6for	AGGAGTCGACACCATGAACACC
CesA6rev	GCTCACAACTGCAGTCGACGGCCC
**secGFP-CesA6 construction**	
Name	Sequences (5'-3')
pCesA6-XhoI	CCCCTCGAGAAAATCAACAAGCAAAATAC
pCesA6-BamHI	GCGGATCCATTTGTCTGAAAACAGACACAGC

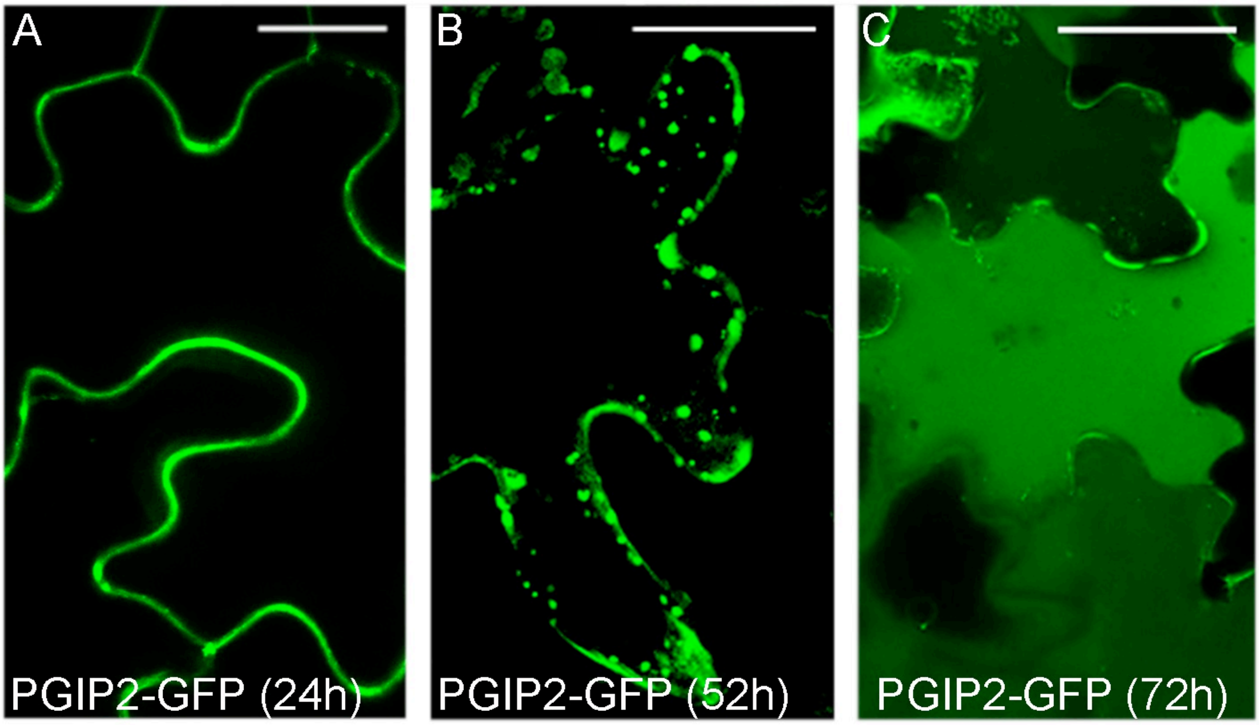

D

Observation time	Cell Wall (%)	Endosomes (%)	Vacuole (%)
24h	93±5	7±5	0
52h	52±8	48±8	0
72h	5±3	23±15	72±17

Supplementary Figure 1. Subcellular localization of PGIP2-GFP transiently expressed in the epidermis of tobacco leaves after agro-infiltration. At 24 hours after infiltration, PGIP2-GFP labeled the cell wall (**A**), at 52 hours after infiltration the fluorescent protein is internalized in endosomes (**B**). After 72 h of PGIP2-GFP expression, it is visible in the large central vacuole and little or nothing in the cell wall (**C**).
Scale bars: 20 µm.
(**D**) Percentage quantification of PGIP2-GFP pattern maturation over time. Definition of classes: Cell Wall was indicated when continuous labeling of cell periphery was observed; Endosomes were indicated when internal compartments and discontinuous labeling of periphery was observed; Vacuole was indicated when central vacuole fluorescent intensity was above 20% of peripheral fluorescence.

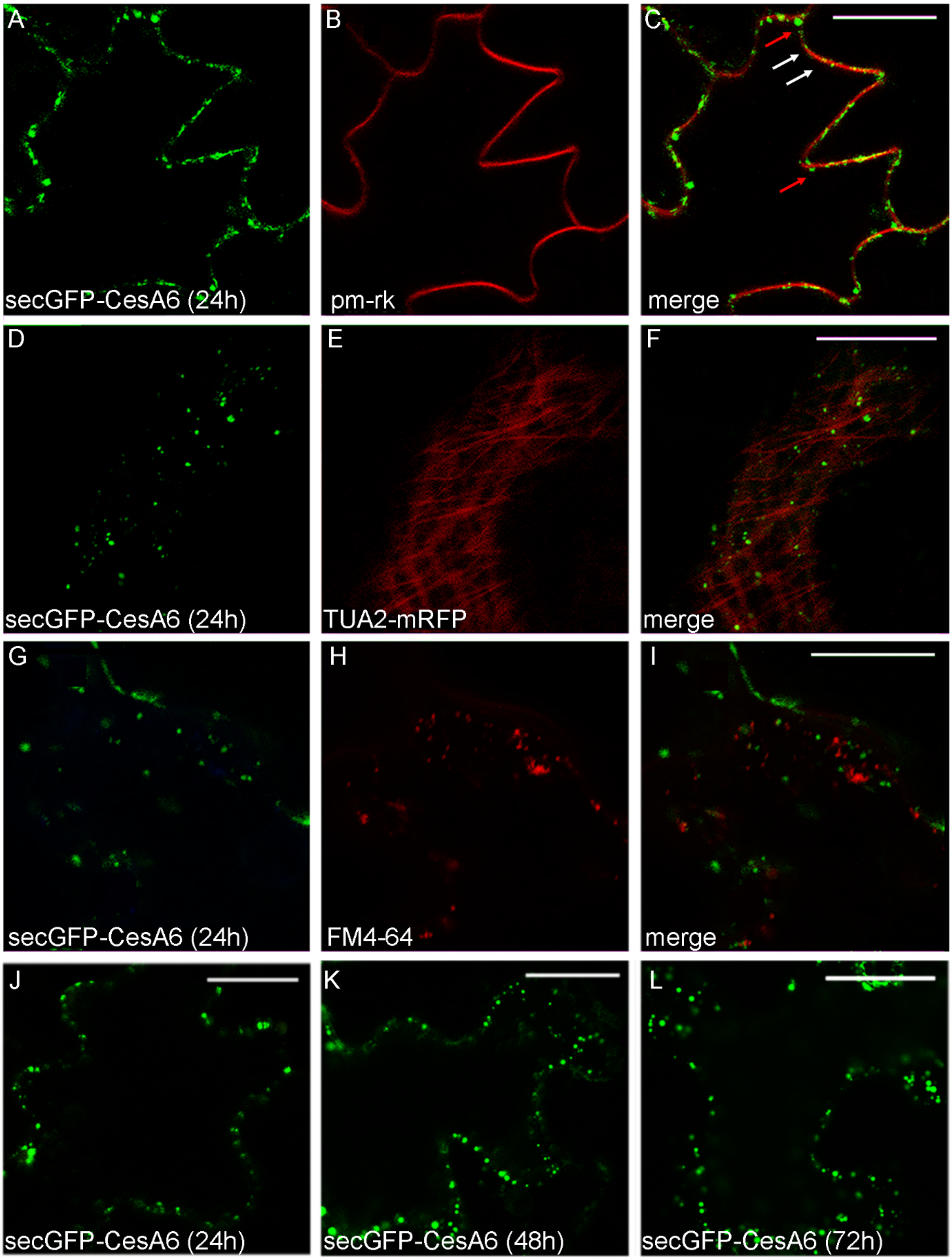

Supplementary Figure 2. Confocal images of tobacco epidermal cells transformed with secGFP-CesA6 (**A**) and pm-rk, plasma membrane marker (**B**). In merged image (**C**), secGFP-CesA6 is localized at the plasma membrane, labeled in red, with a patchy distribution (white arrows) and in intracellular compartments of different sizes (red arrows). Epidermal cells co-transformed with secGFP-CesA6 (**D**) and the microtubule fluorescent marker TUA2-mRFP (**E**) show the close association of MASCs with the microtubules (**F**). The intracellular compartments labeled by secGFP-CesA6 (**G**) and those labeled by FM4-64 (**H**) do not co-localize (**I**). secGFP-CesA6 fluorescent pattern did not change over time (**J**-**L**). Scale bars: 20 µm.

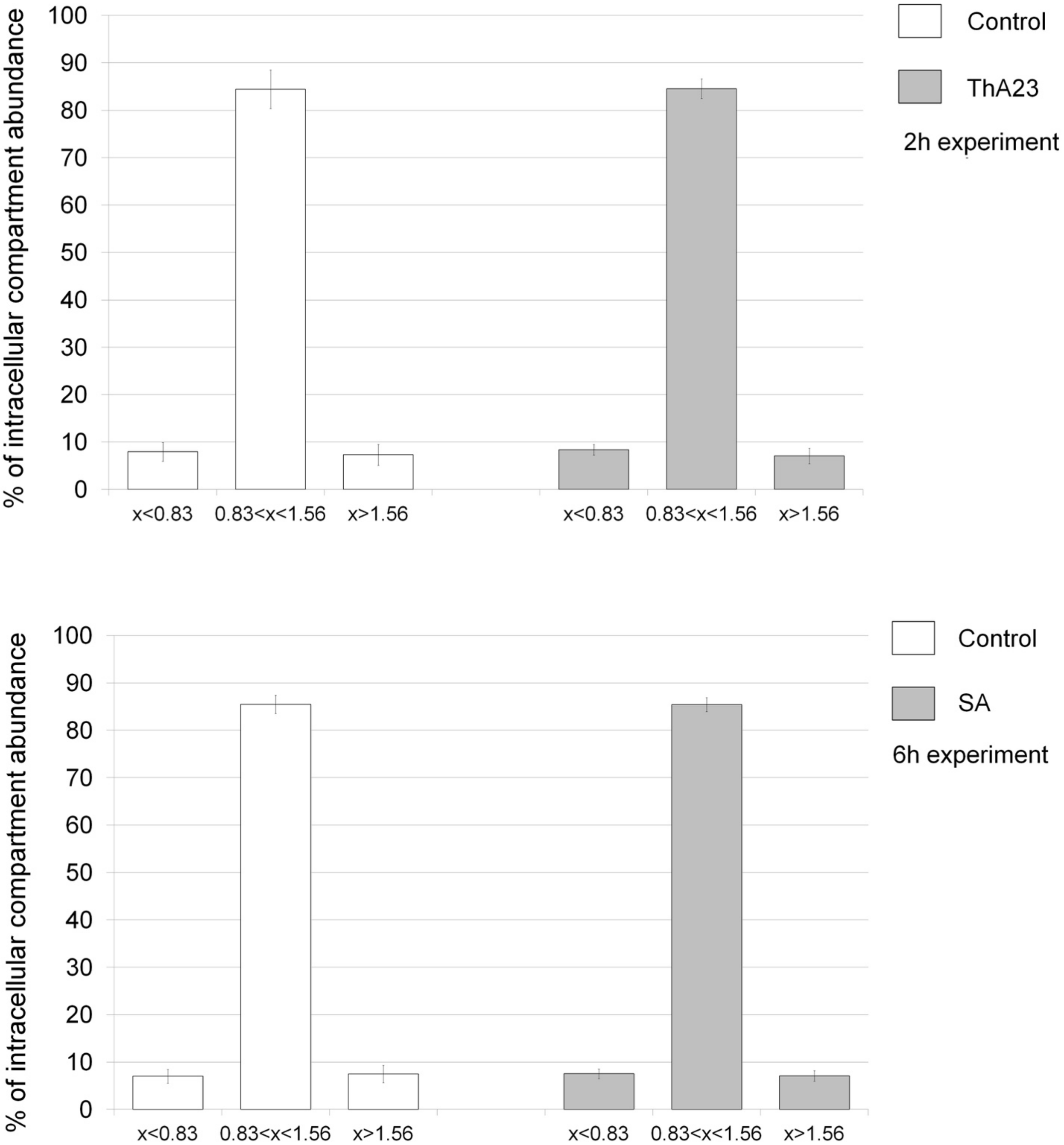

Supplementary Figure 3. Percentage distribution of secGFP-CesA6 labeled compartments classified by size with and without TyrA23 (350 µM) and SA (µM). Three classes of compartments were considered: with dimensions less than 0.83 µm, between 0.83 and 1.56 µm and above 1.56 µm.
P>0.353 for variance significance (one-way ANOVA) between control and corresponding treatments. *n*=12 confocal section from 3 independent experiments. Error bars represent SD.

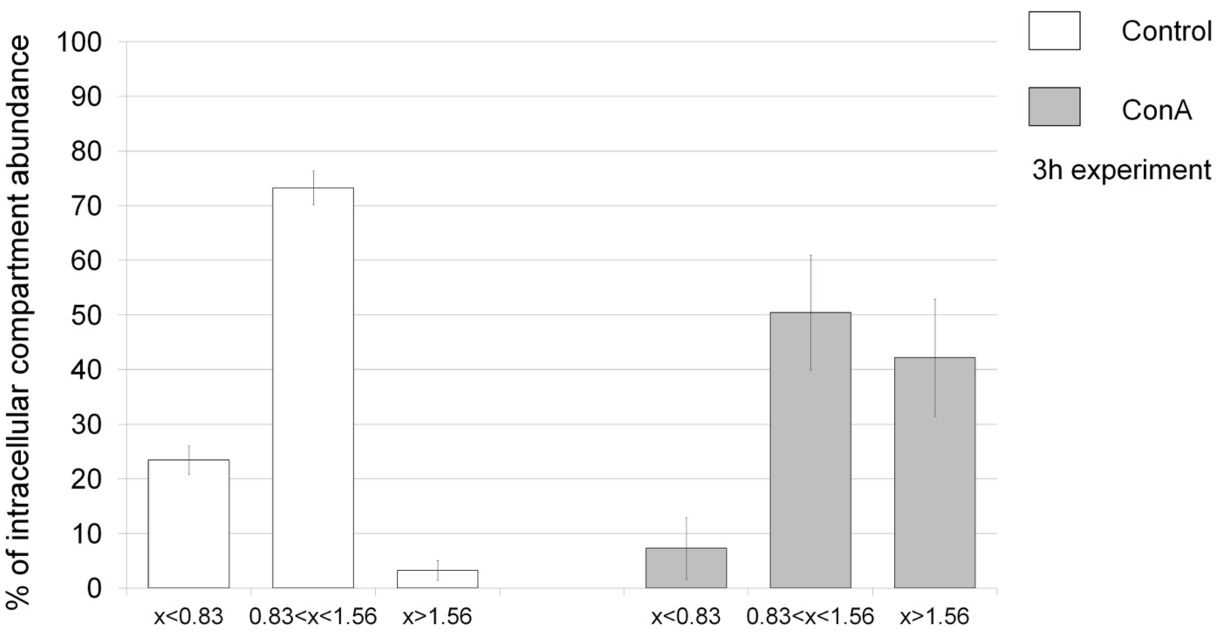

Supplementary Figure 4. Percentage distribution of PGIP2-GFP labeled compartments classified by size with and without ConA (2 µM). Three classes of compartments were considered: with dimensions less than 0.83 µm, between 0.83 and 1.56 µm and above 1.56 µm.

Treatment with ConA (3h) induced significant effects, in fact there is a statistically significant difference between the input groups (P<0.001). *n*=12 confocal section from 3 independent experiments. Error bars represent SD.

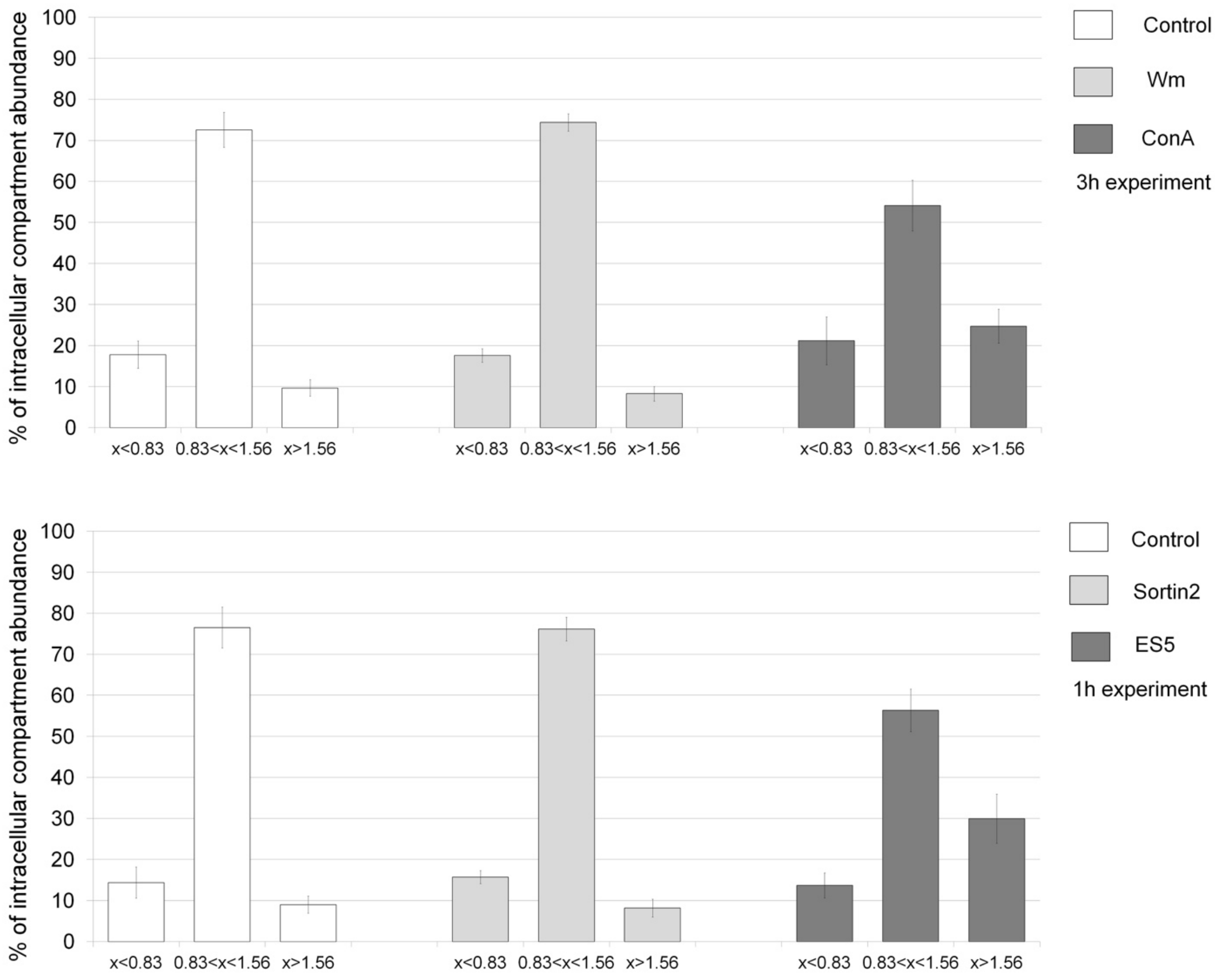

Supplementary Figure 5. Percentage distribution of secGFP-CesA6 labeled compartments classified by size with and without Wm (3 µM), ConA (2 µM), Sortin 2 (20 µM) and ES5 (50 µM). Three classes of compartments were considered: with dimensions less than 0.83 µm, between 0.83 and 1.56 µm and above 1.56 µm.

Treatment with Wm (3h) or Sortin 2 (1h) did not induce significant effects. $P>0.082$ for variance significance (one-way ANOVA) between control and corresponding treatments. Treatment with ConA (3h) or ES5 (1h) induced significant effects ($P<0.001$). $n=12$ confocal section from 3 independent experiments. Error bars represent SD.

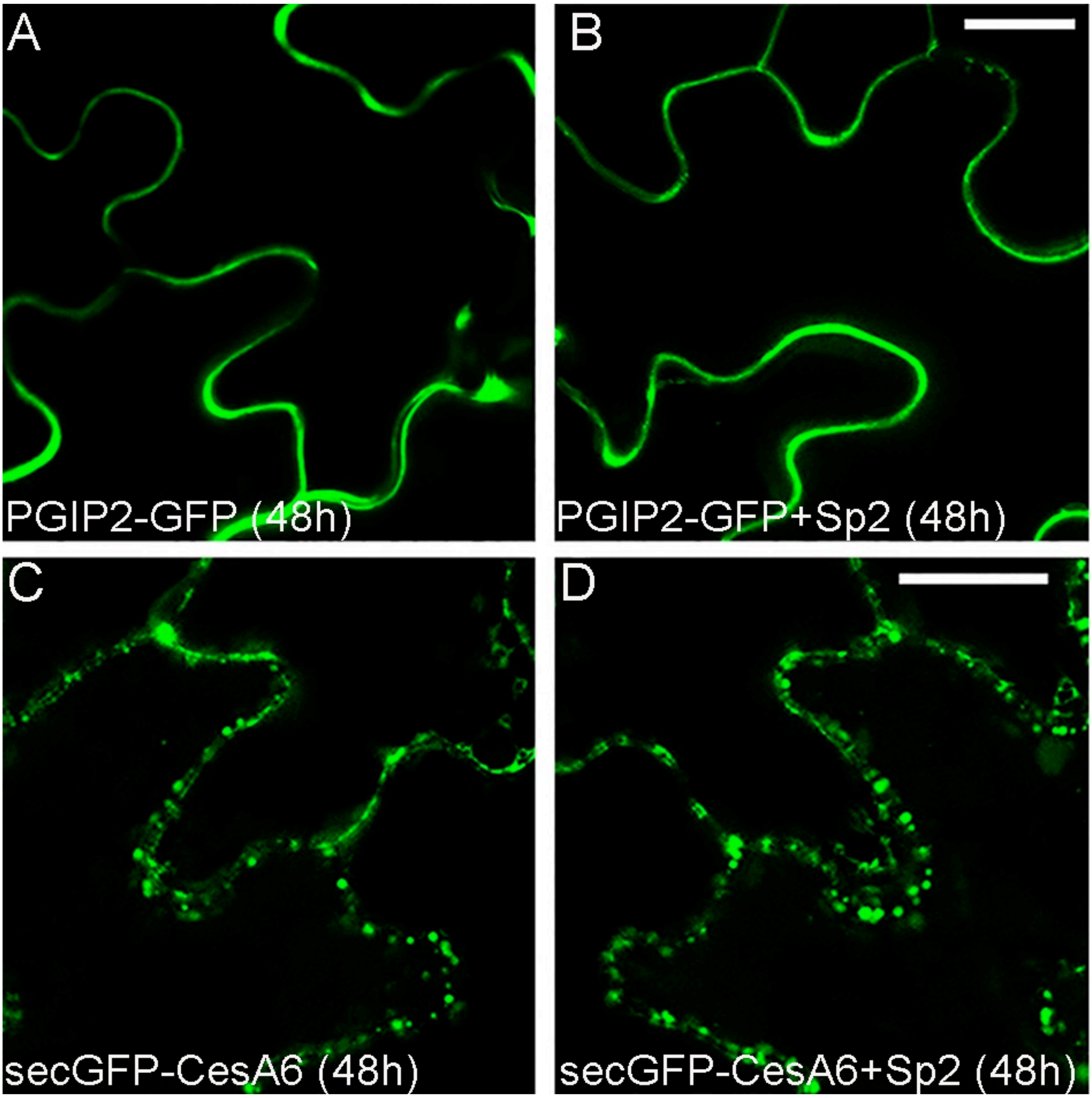

Supplementary Figure 6. Confocal images of epidermal leaves of Sp2 tobacco plants, transformed with PGIP2-GFP and secGFP-CesA6 without **(A, C)** and with **(B, D)** dexamethasone-induced Sp2 expression. Scale bars: 20 µm
